# Neuroprotective and Anti-Inflammatory Activity of *Undaria pinnatifida* Fucoidan In Vivo—A Proteomic Investigation

**DOI:** 10.3390/md23050189

**Published:** 2025-04-27

**Authors:** Cheng Yang, Corinna Dwan, Barbara C. Wimmer, Maurizio Ronci, Richard Wilson, Luke Johnson, Vanni Caruso

**Affiliations:** 1 School of Pharmacy and Pharmacology, University of Tasmania, Hobart, TAS 7005, Australia; cheng.yang@utas.edu.au; 2 Marinova Pty Ltd., 249 Kennedy Drive, Cambridge, TAS 7170, Australia; corinna.dwan@marinova.com.au (C.D.); barbara.wimmer@marinova.com.au (B.C.W.); 3 Department of Medical, Oral and Biotechnology Sciences “G.D’Annunzio”, 66100 Chieti, Italy; maurizio.ronci@unich.it; 4 Central Science Laboratory, College of Science and Engineering, University of Tasmania, Hobart, TAS 7001, Australia; richard.wilson@utas.edu.au; 5 School of Psychological Sciences, Psychology, University of Tasmania, Launceston, TAS 7248, Australia; lukejohnsonphd@gmail.com

**Keywords:** fucoidan, *Undaria pinnatifida*, high-fat diet, anti-inflammatory agents, oxidative stress, neuroprotection, immunomodulation, proteomic analysis, TNF-α, IL-1β, IL-6

## Abstract

*Undaria pinnatifida* fucoidan (UPF), a bioactive sulphated polysaccharide, is widely recognised for its anti-inflammatory, antioxidant, antitumor, anticoagulant, antiviral, and immunomodulatory properties. However, the precise mechanisms by which UPF regulates inflammation and neuronal health remain unclear. This study aimed to investigate the effects of UPF supplementation on pro-inflammatory cytokines in skeletal muscle, small intestine, and the hypothalamus, as well as plasma cytokine levels. Additionally, a brain proteomic investigation in the nucleus accumbens (NAc) was performed to assess UPF’s impact on neuronal protein expression in mice. A total of 64 C57BL/6J mice were administered either a standard chow or high-fat diet (HFD) with or without UPF (400 mg/kg/day) for 10 weeks. In HFD-fed mice, UPF significantly reduced the expression of pro-inflammatory cytokines (TNF-α, IL-1β, and IL-6) in skeletal muscle, small intestine, and hypothalamus, while also lowering circulating IL-1α and IL-6 levels. Proteomic analysis of the NAc revealed that UPF modulated proteins involved in oxidative stress, neuroinflammation, neurotransmitter regulation, and endoplasmic reticulum stress. In contrast, in chow-fed mice, UPF had no effect on the neuroinflammatory–oxidative stress markers but influenced the abundance of proteins associated with immune response and innate immunity. These findings suggest that UPF modulates stress-response pathways in a diet-dependent manner, supporting its potential neuroprotective role in inflammation-related disorders and brain health.

## 1. Introduction

Marine-derived compounds have demonstrated promising neuroprotective effects in preclinical studies, targeting key pathways involved in neuroinflammation and neurodegenerative disorders [1,2]. Among these compounds, fucoidan extracted from *Undaria pinnatifida* (UPF) has gained widespread recognition as a functional food and nutraceutical due to its diverse biological activities and significant contributions to human health. Extensive scientific evidence highlights the potent anticancer [3,4], antioxidant [5], antiarthritic [6] antibacterial [7], and antiviral [8,9] properties of UPF. Additionally, recent studies have demonstrated that UPF can modulate inflammatory pathways by suppressing pro-inflammatory cytokine expression, reducing oxidative stress, and regulating immune cell infiltration in both in vitro and in vivo investigations [6,10,11,12]. However, the precise mechanisms by which UPF exerts its anti-inflammatory effects remain an area of active investigation.

Animal models, particularly rodents, serve as valuable tools for studying inflammation due to their ability to closely mimic human dietary patterns and metabolic responses [13]. Specifically, high-fat diet (HFD) consumption provides critical insights into the complex relationship between inflammation and the mechanisms driving obesity-related metabolic disorders. Rodents consuming HFD develop low-grade, chronic inflammation in adipose tissue, characterised by the infiltration of macrophages and other immune cells, and this condition is consistently linked to elevated systemic inflammation [14,15]. Several investigations have demonstrated that adipocyte hypertrophy, a common consequence of HFD feeding, triggers the release of pro-inflammatory cytokines such as TNF-α, IL-1 β and IL-6, and monocyte chemoattractant protein-1 (MCP-1), promoting chronic low-grade inflammation that affects multiple distant organs including muscle, intestine, and brain [16,17].

Little is known about the effects of UPF supplementation in conjunction with long-term HFD consumption, particularly its influence on inflammatory markers in high-metabolism organs and tissues such as brain, muscles, and the intestine. In our previous investigation, we found that ongoing UPF supplementation enhanced exercise performance, improved muscle function, and positively modulated the gut microbiome in mice, regardless of dietary condition [18]. In vitro studies demonstrated that a 4 h pre-treatment with UPF (10, 50, and 100 µg/mL) significantly suppressed lipopolysaccharide (LPS)-induced upregulation of pro-inflammatory cytokines, including tumour necrosis factor-alpha (TNF-α) and interleukins 1β and 6 (IL-1 β and IL-6), in human macrophages and peripheral blood mononuclear cells [12]. UPF (5–40 μg/mL) also protects hypothalamic neurons from endoplasmic reticulum (ER) stress-induced apoptosis through the Akt/mTOR pathway, highlighting its neuroprotective potential role [19]. In both in vitro and in vivo models of neurodegenerative diseases, UPF exhibited potent antioxidant and neuroprotective effects, significantly reducing amyloid-beta (Aβ1–42) and amyloid-beta (Aβ25–35) aggregation and cytotoxicity in neuronal PC-12 cells while enhancing neurite outgrowth [20,21,22].

In humans, clinical studies further support the anti-inflammatory effects of UPF. One clinical trial showed that a single dose of UPF (1 g) modulated microRNA expression related to immune response and inflammation, highlighting its systemic regulatory potential [23]. In another double-blind randomised placebo-controlled clinical trial, 2 weeks of UPF administration (1 g/day) significantly suppressed the upregulation of inflammatory cytokines induced by high-intensity exercise [24]. Similarly, UPF combined with green-lipped mussel mitigated joint pain and prediabetes, demonstrating its antioxidant and anti-inflammatory effects in a clinical setting [25].

Building on UPF’s known bioactive properties, this study aimed to investigate its potential to counteract the negative effects of HFD consumption on peripheral metabolic organs and in the brain, specifically focusing on its neuroprotective and anti-inflammatory properties.

## 2. Results

### 2.1. Effects of HFD and UPF on Skeletal Muscle Gene Expression

**Figure 1:**
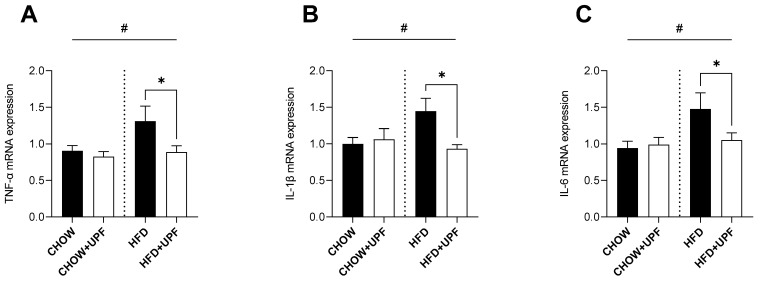
Effects of UPF on skeletal muscle gene expression. mRNA levels of (**A**) TNF-α, (**B**) IL-1β, and (**C**) IL-6. *n* = 11–16. Results are expressed as mean ± SEM. Data were analysed by one-way ANOVA, followed by post hoc LSD tests. #, *p* < 0.05, overall difference between (CHOW and CHOW+UPF) vs. (HFD and HFD+UPF). *, *p* < 0.05, difference between CHOW vs. CHOW+UPF and HFD vs. HFD+UPF.

High-fat diet (HFD) consumption increased the muscular mRNA expression of TNF-α (Figure 1A), IL-1β (Figure 1B), and IL-6 (Figure 1C) compared to a standard chow diet. However, in mice consuming HFD, UPF supplementation significantly downregulated the mRNA expression of TNF-α (−32.2%; *p* < 0.05, Figure 1A), IL-1β (−35.6%; *p* < 0.05, Figure 1B), and IL-6 (−28.9%; *p* < 0.05, Figure 1C). No significant effects of UPF supplementation were observed in the mRNA expression of these cytokines in mice consuming a standard chow diet.

### 2.2. Effects of HFD and UPF on Small Intestine Gene Expression

**Figure 2:**
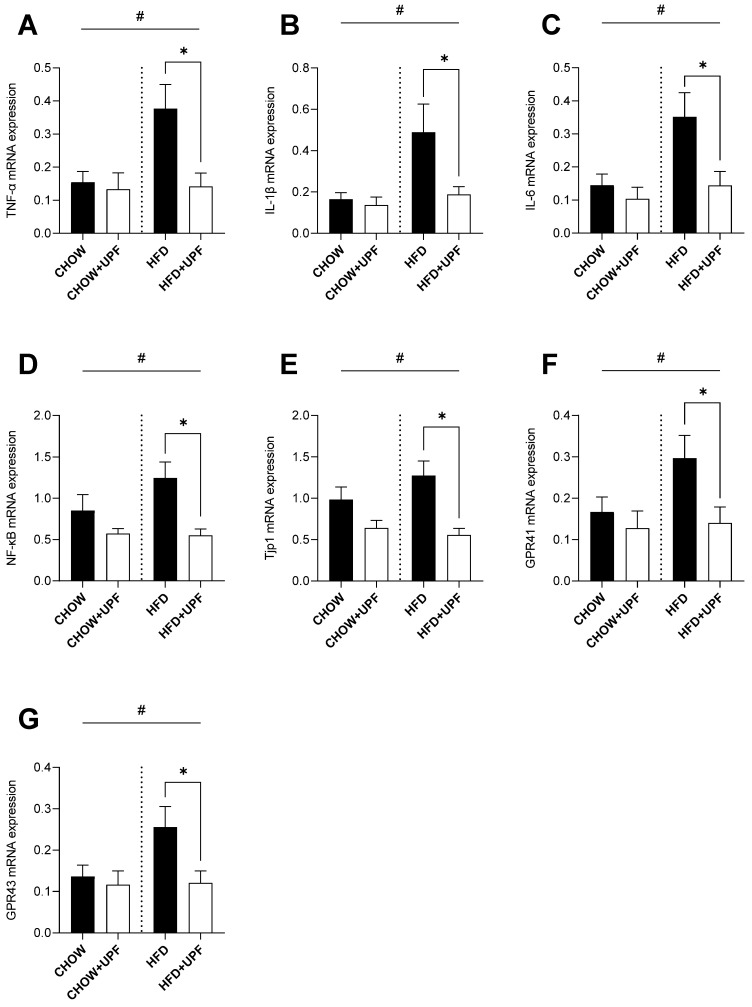
Effects of UPF on small intestine gene expression. mRNA levels of (**A**) TNF-α, (**B**) IL-1β, (**C**) IL-6, (**D**) NF-κB, (**E**) Tjp1, (**F**) GPR41, and (**G**) GPR43. *n* = 7–15. Results are expressed as mean ± SEM. Data were analysed by one-way ANOVA, followed by post hoc LSD tests. #, *p* < 0.05, overall difference between (CHOW and CHOW+UPF) vs. (HFD and HFD+UPF). *, *p* < 0.05, difference between CHOW vs. CHOW+UPF and HFD vs. HFD+UPF.

Overall, HFD increased the intestinal mRNA expression of TNF-α (Figure 2A), IL-1β (Figure 2B), IL-6 (Figure 2C), nuclear factor kappa B (NF-κB, Figure 2D), Tjp1 (Figure 2E), and G-protein coupled receptors 41 and 43 (GPR41 and GPR43, Figure 2F,G) compared to the standard chow diet. However, in mice consuming HFD, UPF supplementation significantly downregulated the mRNA expression of TNF-α (−62.3%; *p* < 0.05, Figure 2A), IL-1β (−62.8%; *p* < 0.05, Figure 2B), IL-6 (−58.9%; *p* < 0.05, Figure 2C), NF-κB (−55.6%; *p* < 0.05, Figure 2D), Tjp1 (−56.5%; *p* < 0.05, Figure 2E), GPR41 (−52.8%; *p* < 0.05, Figure 2F), and GPR43 (−52.9%; *p* < 0.05, Figure 2G). No significant effects of UPF supplementation were observed for these genes in mice consuming a standard chow diet.

### 2.3. Effects of HFD and UPF on Hypothalamic Gene Expression

**Figure 3:**
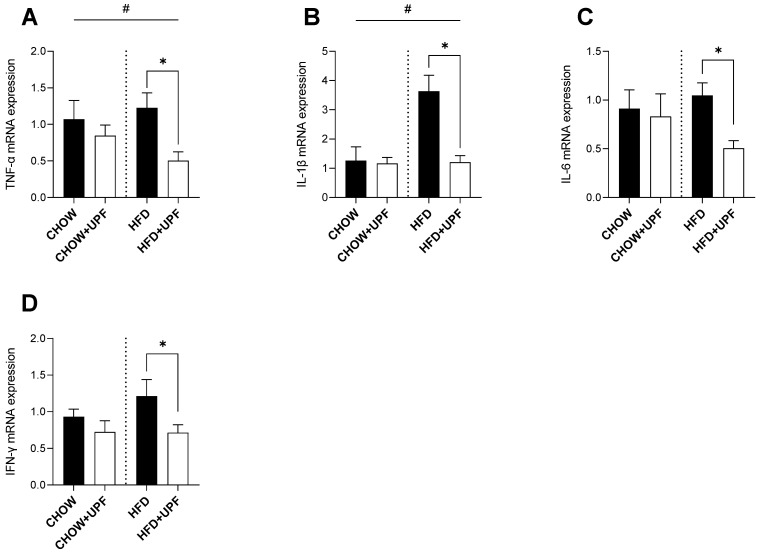
Effects of UPF on hypothalamic gene expression. mRNA levels of (**A**) TNF-α, and (**B**) IL-1β. *n* = 7–10. Results are expressed as mean ± SEM. Data were analysed by one-way ANOVA, followed by post hoc LSD tests. #, *p* < 0.05, overall difference between (CHOW and CHOW+UPF) vs. (HFD and HFD+UPF). *, *p* < 0.05, difference between CHOW vs. CHOW+UPF and HFD vs. HFD+UPF. (**C**) IL-6, and (**D**) IFN-γ. *n* = 7–10. Results are expressed as mean ± SEM. Data were analysed by unpaired *t*-test. *, *p* < 0.05, difference between HFD vs. HFD+UPF.

HFD consumption significantly increased the hypothalamic mRNA expression of TNF-α (Figure 3A) and IL-1β (Figure 3B) compared to a standard chow diet.

In HFD-fed mice, UPF supplementation significantly downregulated the mRNA expression of TNF-α (−58.8%; *p* < 0.05, Figure 3A) and IL-1β (−66.8%; *p* < 0.05, Figure 3B). Additionally, UPF administration significantly reduced the expression levels of IL-6 (−51.9%; *p* < 0.05, Figure 3C) and IFN-γ (−41.2%; *p* < 0.05, Figure 3D) in the hypothalamus. In contrast, UPF supplementation had no statistically significant effect on the mRNA expression of TNF-α (Figure 3A), IL-1β (Figure 3B), IL-6 (Figure 3C), or IFN-γ (Figure 3D) in chow-fed mice.

### 2.4. Effects of HFD and UPF on Pro-Inflammatory Plasma Cytokine Levels

**Figure 4:**
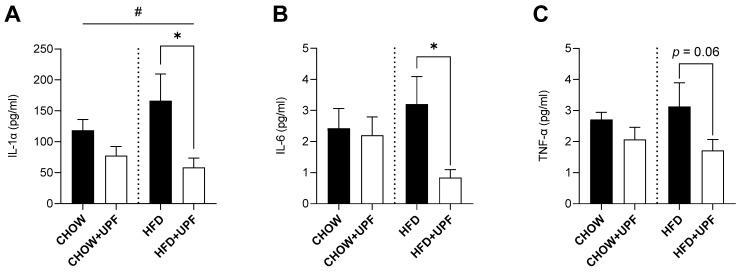
Effects of UPF on plasma cytokines. (**A**) IL-1α. *n* = 6–10. Results are expressed as mean ± SEM. Data were analysed by one-way ANOVA, followed by post hoc LSD tests. #, *p* < 0.05, overall difference between (CHOW and CHOW+UPF) vs. (HFD and HFD+UPF). *, *p* < 0.05, difference between HFD vs. HFD+UPF. (**B**) IL-6, *n* = 4–6. Results are expressed as mean ± SEM. Data were analysed by unpaired *t*-test. *, *p* < 0.05, difference between HFD vs. HFD+UPF. (**C**) TNF-α, *n* = 6–8. Results are expressed as mean ± SEM. Data were analysed by unpaired *t*-test.

HFD consumption elevated the plasma levels of IL-1α (Figure 4A) compared to a standard chow diet. In HFD-fed mice, UPF supplementation significantly decreased IL-1α by 61.7% (*p* < 0.05, Figure 4A), with no statistically significant effect observed in the chow group. Additionally, UPF supplementation significantly downregulated the plasma levels of IL-6 (Figure 4B) by 73.9% (*p* < 0.05, Figure 4B) in mice consuming HFD, while no effect was observed in the chow group.

Although UPF supplementation led to a reduction in TNF-α levels (−45.2%; Figure 4C), this effect was not statistically significant (*p* = 0.06). No statistically significant changes were observed in mice consuming a chow diet.

### 2.5. Effects of UPF on NAc Protein Abundance

**Figure 5:**
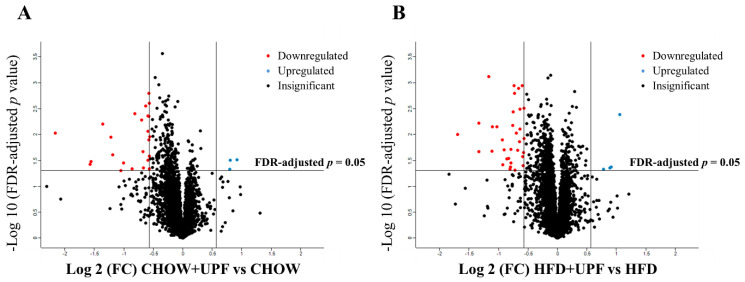
Volcano plot of proteomic changes in mice NAc. (**A**) CHOW+UPF vs. CHOW, and (**B**) HFD+UPF vs. HFD. Proteins that were statistically significantly different relative to the control groups for their respective diets are shown above the horizontal line in red (reduced abundance in the UPF group) or blue (increased abundance in the UPF group).

A total of 5423 proteins were identified in the NAc of mice consuming HFD or a standard chow diet. In the CHOW group, 23 proteins showed significant differences in expression between UPF-treated and control groups (FDR-adjusted *p*-value < 0.05, Figure 5A). In the HFD group, UPF significantly regulated the expression of 36 proteins compared to the control group (FDR-adjusted *p*-value < 0.05, Figure 5B). Volcano plots (Figure 5) highlight these differences, showing proteins with log2 fold changes (FC) greater than ±0.57 and FDR-adjusted *p*-values below 0.05.

#### 2.5.1. Expression Profiles of Differentially Expressed Proteins (DEPs) in the NAc

The differentially expressed proteins (DEPs) are detailed in Table 1 (CHOW+UPF vs. CHOW) and Table 2 (HFD+UPF vs. HFD). Among the 23 DEPs in the CHOW group (Table 1), 3 proteins were upregulated and 20 were downregulated in the NAc of UPF-treated mice. In the HFD group, 4 proteins showed upregulation and 32 downregulation in the NAc of UPF-treated mice (Table 2).

#### 2.5.2. Molecular Functions and Biological Implications of DEPs in the NAc

**Figure 6:**
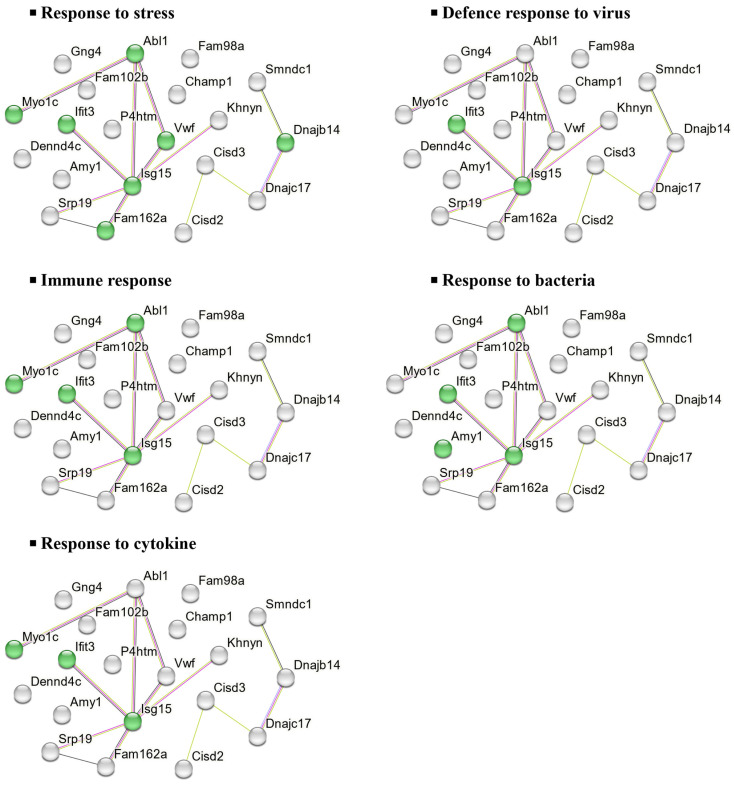
Main biological processes of downregulated DEPs in CHOW groups. PPIs were visualised using the STRING interaction network. Downregulated proteins are represented as a cluster. The coloured proteins in the clusters are involved in the indicated biological process. Classification of proteins was based on Gene Ontology (GO) biological processes. All the biological processes that are referred to present a false-discovery rate of less than 0.05.

To further investigate the molecular functions and biological implications of these DEPs, in silico analysis was performed using STRING, a protein–protein interaction (PPI) network tool. This analysis revealed the key biological processes associated with the 20 downregulated DEPs in the CHOW group, including stress response, viral defence, cytokine response, immune response, and bacterial defence (Figure 6).

**Figure 7:**
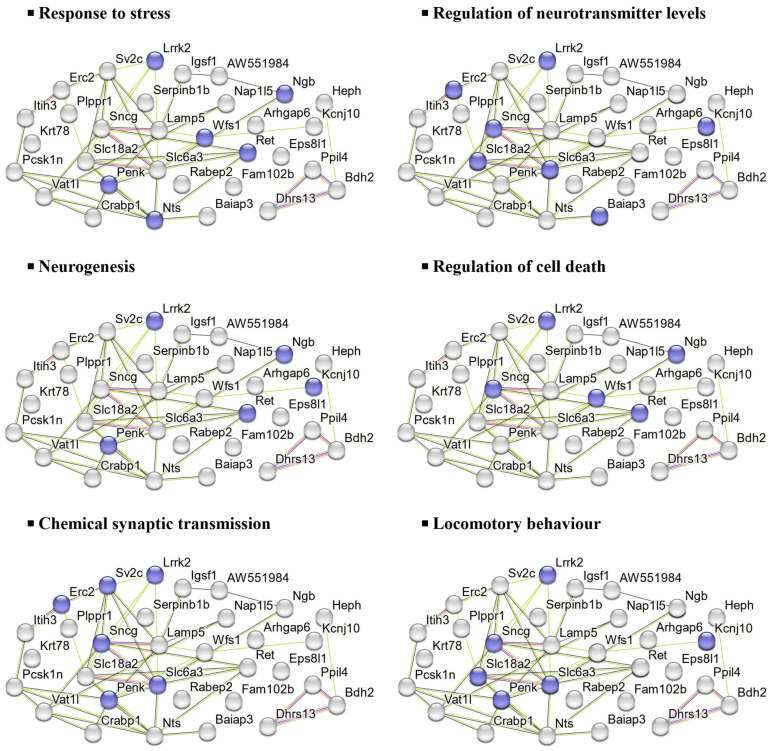
Main biological processes of downregulated DEPs in HFD groups. PPIs were visualised using the STRING interaction network. Downregulated proteins are represented as a cluster. The coloured proteins in the clusters are involved in the indicated biological process. Classification of proteins was based on GO biological processes. All the biological processes that are referred to present a false-discovery rate of less than 0.05.

Similarly, the 32 downregulated DEPs in the HFD group were associated with processes such as stress response, neurotransmitter regulation, neurogenesis, cell death regulation, and synaptic transmission, as shown in Figure 7.

Additionally, in the three upregulated DEPs in the NAc of mice consuming a standard chow diet, Cenpv and Nup210 were associated with cellular component biogenesis and organisation, with no PPI detected in Cops2. In contrast, among the four upregulated DEPs in mice consuming a HFD, Ppm1j and Mlip were involved in cellular metabolic processes. However, no PPI was observed in Fhl2 and Yjefn3.

**Figure 8:**
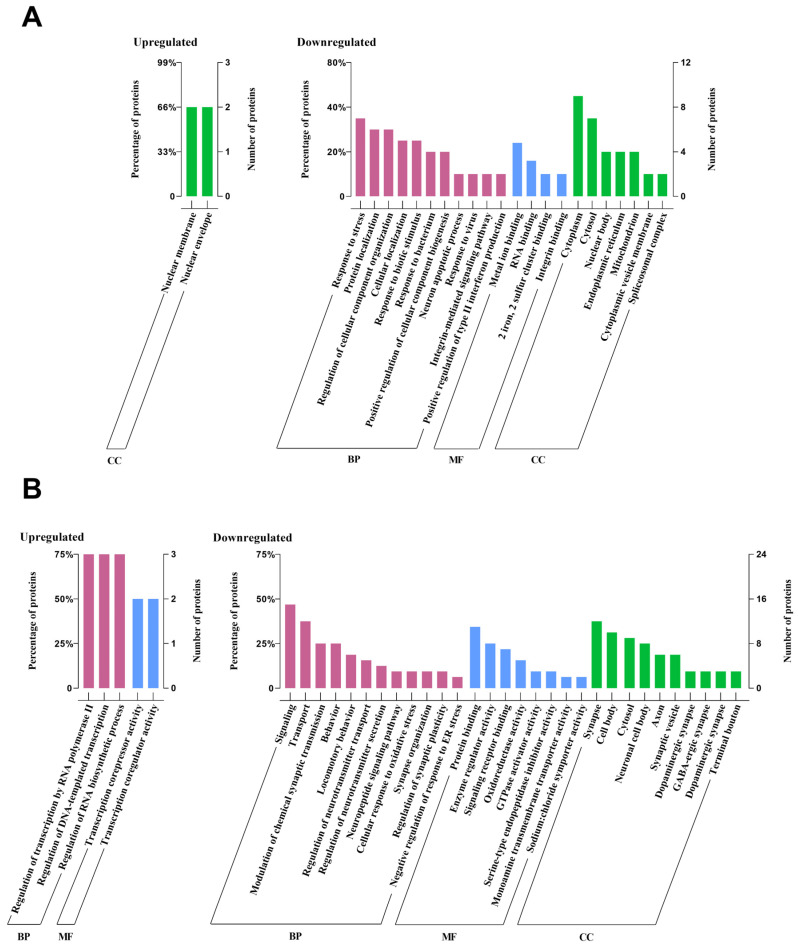
Functional annotation histogram of DEPs. (**A**) CHOW+UPF vs. CHOW, and (**B**) HFD+UPF vs. HFD. Biological process (BP), molecular function (MF), and cellular component (CC) mainly related to the DEPs. The histograms represent the main categories for each GO term in which the upregulated and downregulated DEPs were involved (FDR-adjusted *p*-value < 0.05). The x-axis shows the biological activity, and the y-axis shows the percentage and count of proteins involved in this process among the total proteins in the database.

Furthermore, we carried out a search for biological processes and functions of upregulated and downregulated DEPs by using DAVID. In the CHOW group, the upregulated DEPs were only enriched in the cellular component of the nuclear membrane and nuclear envelope. In contrast, the downregulated DEPs were linked to type II interferon production, stress response, bacterial defence, and iron-sulfur cluster binding (Figure 8A). In the HFD group, the upregulated DEPs were mainly enriched in the biological process of regulation of transcription by RNA polymerase II, DNA-templated transcription, and RNA biosynthetic process, as well as the molecular function of transcription corepressor activity and coregulator activity, while no cellular component was enriched. However, the downregulated DEPs were principally associated with signaling, neurotransmitter transport regulation, oxidative stress, and synapse-related functions (Figure 8B).

## 3. Discussion

This study identified potential mechanisms by which UPF exerts its anti-inflammatory effects in vivo. Specifically, we investigated how the compound modulated plasma pro-inflammatory cytokine protein levels and alterations in gene expression across multiple tissues, such as skeletal muscle, the small intestine, and the hypothalamus. Additionally, we conducted a proteomic analysis of the nucleus accumbens to further investigate UPF brain-specific anti-inflammatory effects and potential neuroprotective activities.

### 3.1. Inflammatory Markers and Gene Expression Studies

Extensive research in animal models indicates that prolonged HFD consumption is typically accompanied by overproduction of pro-inflammatory cytokines including IL-1 and its superfamily members, which results in the activation of the NF-κB pathway, leading to IL-6 and TNF-α secretion [26]. This cascade promotes apoptosis and organ damage and ultimately increases mortality [27,28,29,30,31]. In this study, and consistent with the literature, HFD mice exhibited increased pro-inflammatory cytokine expression (IL-1β, IL-6, TNF-α) in skeletal muscle (Figure 1), the small intestine (Figure 2), and the hypothalamus (Figure 3), while mice consuming standard chow showed no changes in inflammatory markers. Over the 10-week period, UPF supplementation (400 mg/kg/day) significantly reduced plasma levels of IL-1α and IL-6 (Figure 4) as well as mRNA expression of pro-inflammatory cytokines (IL-1β, IL-6, TNF-α) in the skeletal muscle, small intestine, and the hypothalamus of mice consuming HFD (Figure 1, Figure 2 and Figure 3).

Chronic inflammation is a major contributor to muscle wasting and reduced athletic function [32]. Previous investigations on UPF supplementation demonstrated that the compound enhanced exercise performance and improved muscle function in mice, regardless of the diet [18]. In addition, in a clinical trial, acute treatment with a single dose of UPF (1 g) modulated the expression of serum microRNA and affected several biological pathways including inflammation [23]. Our results are also in line with previous research conducted on other species of low-molecular-weight fucoidan [33,34]. Specifically, a 3-week supplementation with *Sargassum hemiphyllum*-derived fucoidan (160 mg/kg/day) reduced NF-κB activation and decreased the expression of pro-inflammatory cytokine (TNF-α, IL-6, and IL-1β) [33], and similarly, a 4-week administration of fucoidan extracted from *Laminaria japonica* (20, 40, and 80 mg/kg/day) protected the gastrocnemius muscles from inflammatory injury in diabetic rats [34].

In the small intestine, several investigations reported that fucoidan extracts showed anti-inflammatory effects by enhancing intestinal barrier function and improving the symptoms of ulcerative colitis while lowering pro-inflammatory cytokine levels [35,36,37,38,39]. In a mouse model of colitis using dextran sodium sulfate (DSS), a chemical colitogen with anticoagulant properties, to induce disease, 28-day oral administration of fucoidan derived from *Scytosiphon lomentaria* (100 and 300 mg/kg/day) inhibited the NF-κB/MAPK pathways, reduced pro-inflammatory cytokine TNF-α, and increased anti-inflammatory IL-10 production [38]. Similarly, in our experiment, suppression of NF-κB activity was accompanied by a reduced TNF-α, IL-1β, and IL-6 expression in mice consuming HFD. In mice consuming a chow diet and treated with UPF, pro-inflammatory cytokine levels were normal.

Tight junction proteins play a crucial role in maintaining the integrity of the intestinal barrier. When dysregulated, they contribute to increased intestinal permeability and chronic inflammation [40,41]. In our study, we observed an upregulation of tight junction protein 1 (Tjp1) in mice fed an HFD. However, UPF supplementation significantly suppressed this dysregulation, suggesting that fucoidan may restore tight junction protein levels to a baseline that supports optimal barrier function helping to maintain intestinal integrity [35,38,42]. Additionally, our study found that UPF treatment inhibited HFD-induced upregulation of GPR 41 and GPR43 in the small intestine. These receptors interact with short-chain fatty acids (SCFAs) such as acetate, propionate, and butyrate—microbial metabolites with a dual role in gut health [43,44]. While SCFA-mediated activation of GPR41 and GPR43 can enhance gut barrier integrity and immune regulation, excessive activation under inflammatory conditions may worsen cytokine production and immune cell recruitment [43,44,45,46,47]. This suggests that UPF-induced downregulation of these receptors could serve as a protective mechanism, preventing hyperactivation and reducing inflammation.

In the hypothalamus of HFD-fed mice, UPF significantly reduced the mRNA expression of pro-inflammatory cytokines, including TNF-α, IL-1, IL-6, and IFN-γ. The hypothalamus is a key regulator of energy balance and is particularly vulnerable to inflammation induced by HFD [48,49]. Hypothalamic inflammation has been associated with disruption of neuronal function and development of systemic metabolic dysfunction [50,51,52]. By suppressing the expression of hypothalamic pro-inflammatory cytokines, UPF may enhance neuronal resilience, further suggesting potential benefits in mitigating diet-induced metabolic disturbances [53,54]. However, further studies are needed to elucidate the mechanisms through which UPF exerts its systemic anti-inflammatory effects, particularly in plasma, skeletal muscle, the small intestine, and the hypothalamus.

To further investigate the anti-inflammatory role of UPF in the brain, we conducted a proteomic analysis of the NAc, a brain region involved in motivation, reward, and addiction [55]. Overall, our findings revealed that UPF modulated the expression of proteins related to immune response, neuronal stability, oxidative and endoplasmic reticulum stress, neurotransmitter regulation, and neuroinflammation, with effects varying based on diet. Specifically, we identified 23 differentially expressed proteins (DEPs) (Table 1) between the CHOW+UPF and CHOW groups (Figure 5A), and 36 DEPs (Table 2) between the HFD+UPF and HFD groups (Figure 5B). For clarity, we structured the discussion of the proteomic results based on the effects of UPF in relation to the specific diet consumed.

### 3.2. Proteomics: Effects of UPF in Mice Consuming Standard Chow

In mice fed a standard chow diet, UPF reduced the abundance of 20 proteins primarily involved in immune responses and cellular stress regulation in the NAc (Figure 6). Regarding immune modulation, two key interferon-stimulated proteins, ubiquitin-like protein interferon-stimulated gene 15 (Isg15) and interferon-induced protein with tetratricopeptide repeats 3 (Ifit3), were downregulated in the NAc of UPF-treated mice. *Isg15*, known for its antiviral and immunomodulatory functions, is induced in CNS cells during immune activation [56,57,58]. Its expression, associated with type I interferons (IFN-α and IFN-β) and other inflammatory mediators (IFN-γ and IL-10), possibly implies a role for UPF in brain immune regulation and anti-inflammatory processes [56,59] (Figure 8A). Likewise, *Ifit3*, which is activated by type I interferons, enhances antiviral defenses and modulates inflammatory pathways [60] (Figure 8A).

UPF also decreased Abl1 (Figure 6), a tyrosine-protein kinase involved in synaptic signaling and neuronal plasticity [61,62]. Overactivation of Abl1 has been linked to neurodegenerative diseases such as Parkinson’s and Alzheimer’s by facilitating α-synuclein accumulation and tau phosphorylation [62,63], suggesting a possible neuroprotective function of UPF in mitigating excitotoxic stress.

Additionally, UPF reduced von Willebrand factor (Vwf) and Myo1c (Figure 6), proteins essential for cerebrovascular stability and cytoskeletal regulation [64,65,66]. While Vwf is critical for hemostasis, it also contributes to vascular inflammation and CNS pathologies [67,68,69]. Myo1c, which stabilizes endothelial cells, prevents excessive Vwf release [66,70]. Their suppression indicates UPF’s potential role in reducing vascular inflammation and preserving neuronal integrity.

Conversely, Cops2 was the most overexpressed protein in UPF-treated, chow-fed mice (Figure 6). As a key component of the COPS complex, it regulates protein degradation, DNA repair, and cellular signaling [71]. Cops2 downregulates Oct-3/4 mRNA, facilitating neuronal differentiation and function [72], while also protecting cells from stress and supporting neuronal health [72,73,74]. Its increased expression points to UPF’s role in enhancing cellular repair and counteracting neurodegeneration associated with DNA damage, though its precise function remains to be fully elucidated.

Similarly, Nup210, the second most overexpressed protein (Figure 6), maintains nuclear–cytoplasmic transport and regulates gene expression [75]. Its elevated levels indicate improved nuclear integrity and transcriptional efficiency, reinforcing neuronal resilience and potentially alleviating neuroinflammation and aging-related impairments [76].

Overall, in mice consuming a chow diet, UPF modulated proteins associated with neuroinflammation, synaptic regulation, and cellular stress responses, highlighting its diverse neuroprotective potential. These findings suggest that UPF may contribute to maintaining brain homeostasis and could play a role in reducing the risk of inflammation-driven neurodegeneration.

### 3.3. Proteomics: Effects of UPF in Mice Consuming HFD

In mice consuming HFD, UPF treatment downregulated 32 proteins linked to oxidative stress responses and apoptosis, neurotransmitter regulation, neurogenesis, synaptic transmission, and behaviour. Among those proteins involved in oxidative stress responses, UPF reduced the abundance of leucine-rich repeat serine/threonine-protein kinase 2 (Lrrk2), wolframin (Wfs1), and neuroglobin (Ngb) (Figure 7).

Lrrk2, a multifunctional protein kinase, is expressed in immune cells like microglia and macrophages, where it regulates inflammatory responses, including the production of pro-inflammatory cytokines [77]. Lrrk2 kinase activates MAPK signalling and increases the production of reactive oxygen species (ROS) and pro-inflammatory cytokines, such as TNF-α, to promote neuroinflammation and apoptosis [77,78,79,80]. Other studies have also reported that mutations in Lrrk2 are linked to oxidative stress conditions and neurodegeneration in PD [77,78].

Wfs1 is a transmembrane protein primarily localised to the endoplasmic reticulum (ER) [81]. Its dysregulation induces ER stress, activating inflammation and apoptotic pathways, and has been linked to β-cell dysfunction, resulting in impaired insulin production and secretion [82]. Additionally, Wfs1 malfunctioning has been associated with neurodegenerative disorders [81,82,83,84].

Our findings showed reduction in levels of Ngb protein, an oxygen-binding globin protein, which has been associated with protection against oxidative stress and neuroprotection by scavenging ROS and supporting oxygen homeostasis [85]. Ngb expression is upregulated under several oxidative stress conditions, such as chronic inflammation, neurodegenerative diseases, and neurotoxin exposure, with its overexpression shown to reduce oxidative damage and improve mitochondrial function in neurons [86,87,88,89]. Mice fed with HFD alone had significantly lower Ngb levels compared to those receiving UPF alongside HFD. This reduction in Ngb under HFD-induced stress might be attributed to the antioxidant property of UPF, which is able to alleviate oxidative stress responses [90,91] (Figure 8B). These findings suggest that UPF might restore neuronal homeostasis and offer potential as a therapeutic strategy for HFD-induced neuroinflammatory and neurodegenerative conditions. Further research is needed to elucidate the precise mechanisms and clinical relevance.

In this study, we found an increase in protein abundance of four and a half LIM domains protein 2 (Fhl2) in the NAc of UPF-treated mice consuming HFD, which suggests significant regulatory effects of UPF on cellular stress responses and inflammatory pathways. Fhl2 plays a significant anti-inflammatory role across various tissues by modulating key pathways like NF-κB and p38 MAPK, balancing immune responses, and promoting tissue repair [92,93,94]. Fhl2 also acts as a scaffolding protein by interacting with various structural and signalling molecules, facilitating cellular processes such as gene transcription, cytoskeletal organisation, and signal transduction [95,96] (Figure 8B). These findings suggest that the ability of UPF to increase Fhl2 protein abundance may underline its neuroprotective and anti-inflammatory properties, highlighting UPF’s potential to modulate cellular and molecular pathways involved in stress adaptation, neurogenesis, and immune regulation.

Similarly, UPF also increased the levels of protein phosphatase 1J (Ppm1j), a member of the metal-dependent Ppm phosphatase family, which possesses key cellular functions such as cell metabolism, oxidative stress response, and immune signalling [97] (Figure 8B). Dysregulation of these phosphatases can lead to abnormal stress responses and metabolic disorders [97]. These findings highlight the potential significance of UPF-induced Ppm1j upregulation in neuronal resilience and stress response; however, additional research is required to uncover the specific mechanisms and functional roles of Ppm1j in brain neurons, particularly in relation to its regulatory pathways and potential implications for neuronal function and neurodegenerative diseases.

### 3.4. Limitations

Future studies should aim to delineate the specific signalling pathways involved in UPF-mediated cytokine modulation, with a particular focus on identifying any direct interactions between UPF and cytokine gene promoters or other elements of the transcriptional machinery. Additionally, extending these investigations to models of chronic inflammation, neurodegeneration, and autoimmune diseases could elucidate the therapeutic scope of UPF in clinical settings. Our study focused on evaluating the intrinsic biological activity of fucoidan without direct comparison to established neuroprotective or anti-inflammatory drugs. Incorporating reference drugs in specifically designed comparative studies could provide further insights into its therapeutic relevance. Moreover, while our study investigated fucoidan’s effects at a specific dose based on the existing literature, exploring multiple doses and their correlation with established data will be essential to fully characterize its anti-inflammatory and neuroprotective potential.

### 3.5. Conclusions

This study provides a descriptive mechanism through which 10 weeks of UPF administration reduced systemic inflammation and supported neuronal health in mice. UPF significantly downregulated pro-inflammatory cytokines (TNF-α, IL-1β, IL-6) in the skeletal muscle, small intestine, and hypothalamus, while lowering circulating IL-1α and IL-6 in HFD-fed mice (Figure 1, Figure 2, Figure 3 and Figure 4). Our findings highlight UPF’s context-dependent effects, modulating distinct protein sets based on metabolic and inflammatory conditions. In chow-fed mice, UPF affected proteins (Isg15, Ifit3, Abl1, Vwf, Myo1c) involved in immune regulation and neuronal stability [56,57,58,59,60,61,62,63,64,65,66,67,68,69,70,71,72,73,74] (Table 1). In HFD-fed mice, UPF influenced stress-response proteins (Lrrk2, Wfs1, Ngb, Slc6a3, Penk) linked to oxidative stress, neuroinflammation, and metabolic dysfunction [85,86,87,88,89,90,91] (Table 2).

Collectively, the findings of this study highlight the diet-dependent effects of UPF, demonstrating its ability to mitigate inflammation and modulate stress-related pathways in HFD-fed mice while exerting distinct immune-related effects in chow-fed mice. The observed changes in cytokine expression and proteomic profiles suggest UPF’s potential neuroprotective support for inflammation-related disorders and brain health, particularly under metabolic stress conditions.

## 4. Materials and Methods

### 4.1. Ethics Statement

This investigation was authorised by the Animal Ethics Committee of the University of Tasmania (A0027164). The animal work and procedures in this study were executed in strict adherence to the provisions delineated in the Tasmanian Animal Welfare Act (1993/63) and the Australian Code of Practice for the Care and Use of Animals for Scientific Purposes 8 edition 2013 [98].

### 4.2. Animals and Diet

Four-week-old male and female C57BL/6J mice (*n* = 32 males and *n* = 32 females, Animal Services, University of Tasmania) were housed under controlled conditions at a temperature of 20 ± 2 °C and maintained on a standard 12:12 h light/dark cycle. Following a one-week acclimatization period, 32 mice were provided with ad libitum access to a commercial high-fat pelleted diet (HFD) (19.4 MJ/kg, 23.5% fat, 23% protein, 5.4% crude fibre, SF16-059, Specialty Feeds, Perth, Australia), while the control group (32 mice) received a standard chow diet (12.8 MJ/kg, 6% fat, 20% protein, 3.2% crude fibre, product code 102108, Ridly Agri-Products) for ten weeks. During this time, mice were singularly housed with unrestricted access to food and drinking water ad libitum.

### 4.3. Fucoidan Administration and Experimental Groups

A fucoidan extract (≥85% fucoidan) derived from *Undaria pinnatifida* (UPF, Batch No.: UPF2022532) was provided by Marinova Pty Ltd. (Tasmania, Australia) and stored at ambient temperature (21–29 °C). The chemical composition of fucoidan used in this study is described below (Table 3 and Table 4).

The chemical composition of fucoidan used in this study is described in the Supplementary Information. Within each dietary cohort, half of the mice received UPF (400 mg/kg/day) in a form incorporated into artificially flavoured and sweetened jelly for voluntary oral administration, while the control group received the vehicle (non-UPF) jelly. Prior to commencing the study, mice were trained to consume the jelly using a previously reported method to ensure they were accustomed to its taste and texture [99]. The successful use of this method has already been described elsewhere [18]. This method yielded four experimental groups (*n* = 16 per group): CHOW, CHOW+UPF, HFD, and HFD+UPF. Dietary and UPF intervention continued for ten weeks.

### 4.4. Sample Collection

Mice were fasted overnight (12 h) and euthanised via carbon dioxide inhalation for plasma and tissue collection. Blood samples were collected in a 1.5 mL Eppendorf tube containing 7.5 µL of Heparin (1000 U/mL) by cardiac puncture and kept on ice. Plasma supernatants were collected by centrifugation (12,000 rpm/10 min, R.T.). Plasma was aliquoted and stored at −80 °C until use. Plasma levels of inflammatory cytokines IL-1α, IL-6, and TNF- α were measure with a commercial MILLIPLEX^®^ mouse high-sensitivity T cell magnetic bead panel (MHSTCMAG-70K, Millipore, Cannon Hill, Australia) according to the manufacturer’s instructions. Mice small intestines, skeletal muscles (gastrocnemius), hypothalamus, and NAc were dissected, snap-frozen in liquid nitrogen, and stored at −80 °C for RNA extraction in gene expression studies and for protein extraction in proteomic assays.

### 4.5. Real-Time Quantitative PCR (RT-qPCR) Assay

Total mRNA was extracted from the mice’s small intestine, skeletal muscle, and hypothalamus tissues using an RNeasy Mini Kit (Cat# 74106, Qiagen, Chuo-ku, Japan) according to the manufacturer’s recommendations. A Nanodrop^TM^ 8000 Spectrophotometer (NanoDrop Technologies Inc., Minato-ku, Japan) was used to measure the RNA concentration and purity ratios (A260/280 and A260/230), and only samples with an absorbance ratio of ~2.0 were used for complementary DNA (cDNA) synthesis. One microgram of RNA template from each sample was reverse-transcribed into cDNA using a High-Capacity cDNA Reverse Transcription Kit (Cat# 4368814, Applied Biosystem, Waltham, MA, USA). All primers were purchased from Kicqstart SYBR Green Primers (Sigma-Aldrich, St. Louis, MO, USA). The sequence of the primers used for quantification of gene expression of TNF-α, IL-1β, IL-6, IL-10, NF-κB, Tjp1, GPR41, GPR43, TLR4, *β-actin*, and *GAPDH* can be found in the Supplementary Information. RT-qPCR reactions were performed using PowerUp SYBR green master mix (Cat# A25918, Applied Biosystems, Waltham, MA, USA) in the CFX Connect TM Real-Time PCR Detection system (Bio-Rad, South Granville, Australia) following the manufacturer’s instructions. The amplification program included an initial denaturation step at 95 °C for 5 min, followed by 40 cycles of 10 s at 95 °C, 10 s at 60 °C, and 20 s at 72 °C. After each amplification, a melting curve study was conducted to validate the reliability of the results and product specificity. Target gene expression was normalised against *β-actin* and *GAPDH* housekeeping genes using the average of control group as a calibrator. Analysis was performed using the comparative 2^−ΔΔCt^ method [100]. Samples were included in the RT-qPCR analysis only if they met the quality control criteria, which involved RNA integrity assessment (RIN) ≥ 7.0, cDNA synthesis efficiency, and proper amplification performance. Samples exhibiting poor RNA quality, inconsistent reverse transcription efficiency, irregular amplification curves, or statistical outliers (identified using Grubbs’ test) were excluded from the final analysis. As a result, the number of biological replicates (n) varied across different experiments, reflecting these quality control measures [18,50,101,102,103].

### 4.6. Proteomic Analysis

Frozen NAc tissues were homogenised using a tissue protein extraction reagent (TPER, Thermo Fisher Scientific, Rockford, IL, USA) to efficiently lyse cells and solubilise proteins. Protein concentrations in the extracts were quantified using a Nanodrop Spectrophotometer (NanoDrop Technologies Inc., Minato-ku, Japan) at an absorbance at A660 nm to ensure uniform protein (30 μg/90 μL) quantities for downstream analysis.

Proteins were reduced with 10 mM dithiothreitol (DTT) overnight at 4 °C, alkylated with 55 mM iodoacetamide (IAA) for 2 hrs in the dark at room temperature, and then digested with 1.2 μg trypsin/rLysC (Promega, Madison, WI, USA) after sample clean-up according to the SP3 method [104]. Peptides were then desalted using C18 ZipTips (Millipore, Billerica, MA, USA) and analysed by nano-flow HPLC and data-independent acquisition mass spectrometry using a Q-Exactive HF and Ultimate 3000 RSLCnano, as previously described [105].

### 4.7. Proteomics Data Processing and Statistical Analysis

Raw DIA-MS files were imported into Spectronaut software version 18 (Biognosys) and searched against the *Mus musculus* UniProt proteome UP00000589 comprising 44,456 entries (accessed on 6 April 2021) using the Pulsar search engine according to Biognosys (BGS) factory settings. The resulting library of 37,622 proteotypic peptides, mapped to 6298 protein groups, was then used for targeted data re-extraction and cross-run protein quantitation/normalisation using BGS factory settings, with the exception that single-hit protein IDs were excluded. The Perseus software platform (version 2.0.11) was used for imputation of missing intensity values, statistical analysis, and data visualization.

Differentially abundant proteins were identified using pair-wise *t*-test comparisons of all replicates (*n* = 10) of both CHOW and HFD (CHOW vs. CHOW+UPF and HFD vs. HFD+UPF), with a false-discovery rate (FDR) of 0.05 and an s0 value of 0.1 used to define significant proteins. Only proteins with a statistically significant change (as defined by FDR ≤ 0.05) and a moderated *t*-test incorporating an s0 value of 0.1 were reported as differentially expressed. Functional analyses of the differentially abundant proteins were performed using DAVID (Database for Annotation Visualization and Integrated Discovery) version 2024 (https://david.ncifcrf.gov/, accessed on 12 September 2024) [106,107] to analyse biological processes, molecular functions, and cellular components. The STRING functional protein association network (https://string-db.org/, accessed on 12 September 2024) [108] was used to develop interaction maps.

Other statistical analyses were performed using GraphPad Prism version 8.3.0 for Windows (GraphPad Software, San Diego, CA, USA, www.graphpad.com, accessed on 12 September 2024), and results were expressed as mean ± SEM. The effects of UPF on mRNA gene expression in mice muscle, intestine, and hypothalamus, and plasma inflammatory cytokine levels were analysed by a one-way ANOVA. ANOVA results were then assessed by a post hoc analysis using Fisher’s least significant difference test (LSD) as appropriate. When no overall diet effect was observed between groups [(CHOW and CHOW+UPF) vs. (HFD and HFD+UPF)], data were analysed using an unpaired *t*-test. Results were considered statistically significant when *p* < 0.05.

## Figures and Tables

**Table 1:** Proteins with significantly differential expression between the CHOW+UPF and CHOW group using Perseus software platform.

Protein	UniProt Accession	Gene Name	FDR	FC
COP9 signalosome complex subunit 2	A2AQE4	Cops2	0.030	1.9
Nuclear pore membrane glycoprotein 210	Q9QY81	Nup210	0.031	1.8
Centromere protein V	Q9CXS4	Cenpv	0.047	1.7
Interferon-induced protein with tetratricopeptide repeats 3	Q64345	Ifit3	0.004	0.7
DnaJ homolog subfamily C member 17	Q91WT4	Dnajc17	0.002	0.7
Signal recognition particle 19 kDa protein	Q9D104	Srp19	0.013	0.7
DENN domain-containing protein 4C	A6H8H2	Dennd4c	0.032	0.7
Survival of motor neuron-related-splicing factor 30	Q8BGT7	Smndc1	0.009	0.7
CDGSH iron–sulfur domain-containing protein 2	Q9CQB5	Cisd2	0.004	0.7
Protein FAM102B	Q8BQS4	Fam102b	0.003	0.6
CDGSH iron–sulfur domain-containing protein 3, mitochondrial	B1AR13	Cisd3	0.044	0.6
Guanine nucleotide-binding protein G(I)/G(S)/G(O) subunit gamma-4	P50153	Gng4	0.021	0.6
Protein KHNYN	Q80U38	Khnyn	0.005	0.6
Alpha-amylase 1	P00687	Amy1	0.046	0.6
Ubiquitin-like protein ISG15	Q64339	Isg15	0.036	0.5
DnaJ homolog subfamily B member 14	Q149L6	Dnajb14	0.050	0.5
Chromosome alignment-maintaining phosphoprotein 1	Q8K327	Champ1	0.025	0.4
Protein FAM162A	Q9D6U8	Fam162a	0.011	0.4
Tyrosine-protein kinase ABL1	P00520	Abl1	0.006	0.4
Transmembrane prolyl 4-hydroxylase	Q8BG58	P4htm	0.034	0.3
Unconventional myosin-Ic	Q9WTI7	Myo1c	0.037	0.3
von Willebrand factor	Q8CIZ8	Vwf	0.009	0.2
Protein FAM98A	Q3TJZ6	Fam98a	0.015	0.2

Fold changes (FCs) shown in upregulated proteins if FC > 1 or in downregulated proteins if FC < 1. Sample size: *n* = 10 in both CHOW+UPF and CHOW Groups.

**Table 2:** Proteins with significantly differential expression between the HFD+UPF and HFD group using Perseus software platform.

Protein	UniProt Accession	Gene Name	FDR	FC
Protein phosphatase 1J	Q149T7	Ppm1j	0.004	2.1
Four and a half LIM domains protein 2	O70433	Fhl2	0.043	1.9
YjeF N-terminal domain-containing protein 3	F6W8I0	Yjefn3	0.044	1.9
Muscular LMNA-interacting protein	V9GWW6	Mlip	0.047	1.7
Rho GTPase-activating protein 6, isoform CRA_b	G3UZI7	Arhgap6	0.012	0.7
Synaptic vesicle glycoprotein 2C	Q69ZS6	Sv2c	0.022	0.7
Sodium-dependent dopamine transporter	Q61327	Slc6a3	0.040	0.7
Dehydrogenase/reductase SDR family member 13	Q5SS80	Dhrs13	0.026	0.7
Gamma-synuclein	Q9Z0F7	Sncg	0.001	0.7
Hephaestin	Q9Z0Z4	Heph	0.003	0.6
Wolframin	Q3UN10	Wfs1	0.008	0.6
ATP-sensitive inward rectifier potassium channel 10	Q9JM63	Kcnj10	0.014	0.6
Lipid phosphate phosphatase-related protein type 1	Q8BFZ2	Lppr1	0.001	0.6
Epidermal growth factor receptor kinase substrate 8-like protein 1	Q8R5F8	Eps8l1	0.020	0.6
Lysosome-associated membrane glycoprotein 5	Q9D387	Lamp5	0.009	0.6
Synaptic vesicular amine transporter	Q8BRU6	Slc18a2	0.049	0.6
Inter-alpha-trypsin inhibitor heavy chain H3	Q61704	Itih3	0.002	0.6
Nucleosome assembly protein 1-like 5	Q9JJF0	Nap1l5	0.001	0.6
ProSAAS	Q9QXV0	Pcsk1n	0.004	0.6
Synaptic vesicle membrane protein VAT-1 homolog-like	Q80TB8	Vat1l	0.007	0.6
Leucine-rich repeat serine/threonine-protein kinase 2	Q5S006	Lrrk2	0.036	0.6
Proto-oncogene tyrosine-protein kinase receptor Ret	P35546	Ret	0.019	0.6
Peptidyl-prolyl cis-trans isomerase-like 4	Q9CXG3	Ppil4	0.042	0.6
ERC protein 2	Q6PH08	Erc2	0.029	0.6
Proenkephalin-A	P22005	Penk	0.029	0.6
Cellular retinoic acid-binding protein 1	P62965	Crabp1	0.020	0.5
Protein FAM102B	Q8BQS4	Fam102b	0.038	0.5
Protein AW551984	Q8BGF0	AW551984	0.013	0.5
3-hydroxybutyrate dehydrogenase type 2	Q8JZV9	Bdh2	0.007	0.5
Leukocyte elastase inhibitor B	Q8VHP7	Serpinb1b	0.007	0.5
BAI1-associated protein 3	S4R1E7	Baiap3	0.021	0.5
Neuroglobin	Q9ER97	Ngb	0.001	0.4
Protein Krt78	E9Q0F0	Krt78	0.021	0.4
Neurotensin/neuromedin N	Q9D3P9	Nts	0.006	0.4
Immunoglobulin superfamily member 1	Q7TQA1	Igsf1	0.010	0.3
Rab GTPase-binding effector protein 2	Q91WG2	Rabep2	0.028	0.1

Fold changes (FCs) shown in upregulated proteins if FC > 1 or in downregulated proteins if FC < 1. Sample size: *n* = 10 in both HFD+UPF and HFD groups.

**Table 3:** Absolute mass percentages of *Undaria pinnatifida* fucoidan (UPF) extract.

Fucoidan Extract	Neutral Carbohydrates (%)	Sulfate (%)	Fucoidan (%)	Polyphenols (%)
UPF2022532	46.1	28.3	89.3	<2

**Table 4:** Carbohydrate breakdown (mass %) of neutral carbohydrates in *Undaria pinnatifida* fucoidan (UPF) extract.

Fucoidan Extract	Fucose (%)	Xylose (%)	Galactose (%)	Arabinose (%)	Rhamnose (%)
UPF2022532	22.5	0.3	19	0.6	0.6

## Data Availability

The original data presented in the study are openly available in the ProteomeXchange Consortium via the PRIDE partner repository (https://www.ebi.ac.uk/pride/) [109] with the dataset identifier PXD06086 [accessible via login reviewer_pxd060861@ebi.ac.uk; password 7Mwe5q83hJPo].

## Supplementary Materials

The following supporting information can be downloaded at: https://www.mdpi.com/article/10.3390/md23050189/s1. Table S1: Sequence of primers used for RT-qPCR assay in mouse skeletal muscle, small intestine, and hypothalamus.
