# Supplementary material for: Neuroprotective and Anti-Inflammatory Activity of Undaria pinnatifida Fucoidan In Vivo—A Proteomic Investigation
Source: Mar Drugs. 2025 Apr 27;23(5):189. doi: 10.3390/md23050189 (PMC12113125; doi:10.3390/md23050189)
Supplement: Supplementary file 1 [file marinedrugs-23-00189-s001.zip › marinedrugs-3560895-supplementary.pdf]

Supplementary Information

Table S1. Sequence of primers used for RT-qPCR assay in mouse skeletal muscle, small intestine, and hypothalamus.

Gene	Forward primer (5' to 3')	Reverse primer (3' to 5')
IL-1β	TGGACCTTCCAGGATGAGGACA	GTTCATCTCGGAGCCTGTAGTG
TNF-α	GGACTAGCCAGGAGGGAGAACAG	GCCAGTGAGTGAAAGGGACAGAA
IL-6	CGGAGAGGAGACTTCACAGAGGA	TTTCCACGATTTCCCAGAGAACA
NF-κB	CCTCTGGCGAATGGCTTTAC	GCTATGGATACTGCGGTCTGG
IFN-γ	GGATGCATTCATGAGTATTGC	CTTTTCCGCTTCCTGAGG
Tjp1	CTCCGATCATTCCACGCAGT	TTCGGTTCTGGAAGAGTGGG
GPR41	TTGCTAAACCTGACCATTTCGG	GATAGGCCACGCTCAGAAAAC
GPR43	ACAGTGGAGGGGACCAAGAT	GGGGACTCTCTACTCGGTGA
Housekeeping		
β-actin	CCTAAGGCCAACCGTGAAAA	AGCCATACAGGGACAGCACA
GAPDH	AAGGTCGGTGTGAACGGATTTG	TGTAGACCATGTAGTTGAGGTCA
